# Short-Term Active Safety Surveillance of the Spikevax and Nuvaxovid Priming Doses in Australia

**DOI:** 10.3390/vaccines12090971

**Published:** 2024-08-27

**Authors:** Renee Reynolds, Evelyn Tay, Michael Dymock, Lucy Deng, Catherine Glover, Laura K. Lopez, Yuanfei Anny Huang, Patrick Cashman, Alan Leeb, Julie A. Marsh, Tom Snelling, Nicholas Wood, Kristine Macartney

**Affiliations:** 1 Population Health, Hunter New England Local Health District, Wallsend, NSW 2287, Australia; 2 School of Medicine and Public Health, College of Health, Medicine and Wellbeing, The University of Newcastle, Callaghan, NSW 2308, Australia; 3 Hunter Medical Research Institute, New Lambton Heights, NSW 2305, Australia; 4 Wesfarmers Centre of Vaccines and Infectious Diseases, Telethon Kids Institute, Nedlands, Perth, WA 6009, Australia; 5 National Centre for Immunisation Research and Surveillance, Westmead, Sydney, NSW 2145, Australia; 6 The Children’s Hospital at Westmead, Westmead, Sydney, NSW 2145, Australia; 7 Faculty of Medicine and Health, The University of Sydney, Camperdown, Sydney, NSW 2050, Australia; 8 Centre for Child Health Research, University of Western Australia, Nedlands, Perth, WA 6009, Australia; 9 Illawarra Medical Centre, Ballajura, Perth, WA 6066, Australia

**Keywords:** COVID-19, vaccine, safety, active surveillance, adverse events, Moderna, Novavax

## Abstract

Australia commenced administration of the Spikevax (Moderna mRNA-1273) COVID-19 vaccine in August 2021 and Nuvaxovid (Novavax NVX-CoV2373) in January 2022. This study describes the short-term safety profile of priming doses of the Spikevax and Nuvaxovid vaccines given between September 2021 and September 2023. Online surveys were sent via AusVaxSafety, Australia’s active vaccine safety surveillance system, three and eight days after vaccination. A total of 131,775 day 3 surveys were sent, with a response rate of 38.5% (*N* = 50,721). A total of 43,875 day 8 surveys matched with day 3 survey responses were sent, with a response rate of 71.5% (*N* = 31,355). Half (50.7%) of respondents reported any adverse event following immunisation (AEFI) in the 0–3 days after vaccination and 24.6% reported any AEFI 4–7 days after vaccination. Fatigue, local pain, headache, and myalgia were the most frequently reported symptoms for both vaccines in both periods. After adjusting for respondent characteristics, vaccination clinic type, jurisdiction, and medical conditions, the odds for reporting AEFI increased with age from 16–19 years to highest odds at 30–39 years, after which it declined. Females had greater odd of reporting AEFI than males across most age groups, vaccine types, and doses. Respondents with a history of anaphylaxis had greater odds of reporting any AEFI (adjusted OR range: 1.50–2.86). A total of 3.1% of respondents reported seeking medical review 0–3 days after vaccination. This study affirms the short-term safety of Spikevax and Nuvaxovid COVID-19 vaccine priming doses in a large sample in Australia.

## 1. Introduction

Australia approved four vaccines for use as primary doses during the COVID-19 vaccination program: Comirnaty (Pfizer–BioNTech BNT162b2), Vaxzevria (AstraZeneca ChAdOx1), Spikevax (Moderna mRNA-1273) and Nuvaxovid (Novavax NVX-CoV2373). The short-term safety of Comirnaty and Vaxzevria primary doses (the initial vaccine brands available in Australia) was reported by Deng et al. [1] using AusVaxSafety active, national surveillance in the Australian population from February to August 2021.

Spikevax, a lipid-nanoparticle encapsulated mRNA vaccine, was approved for use in Australia by the Therapeutic Goods Administration (TGA) as a primary course for individuals aged 18 years and over on 9 August 2021, 12–17 years on 3 September 2021, 6–11 years on 17 February 2022, and 6 months to 5 years on 19 July 2022 [2]. The Nuvaxovid recombinant spike protein adjuvanted vaccine was provisionally approved by the TGA as a primary course for individuals aged 18 years and older on 19 January 2022 and 12–17 years on 22 July 2022 [2]. The approval of Nuvaxovid provided an alternative vaccine option, particularly for people who had previously experienced an adverse event following immunisation (AEFI) after receiving an mRNA- (Comirnaty or Spikevax) or adenovirus vector-based (Vaxzevria) [3,4] COVID-19 vaccine or those who were hesitant to receive an mRNA- or adenovirus vector-based vaccine (potentially due to concerns over perceived ‘new’ technologies [5] or serious AEFI such as myocarditis).

Clinical trials provide critical data on the reactogenicity and immunogenicity/efficacy of vaccines for regulatory approval but are conducted in limited populations and time periods. Post-licensure monitoring of vaccines allows for near real-time surveillance of AEFI in whole populations, including individuals with comorbidities and concurrent administration of vaccines, contributing to immunisation provider confidence in vaccines and providing ongoing safety data to regulatory agencies [6]. Led by the National Centre for Immunisation Research and Surveillance (NCIRS), AusVaxSafety is Australia’s active vaccine safety surveillance system, monitoring the post-licensure safety of vaccines in Australia since 2014 [7]. AusVaxSafety actively collects information on solicited and unsolicited AEFI, whether medical review was sought, and impact on usual activities following vaccination. Rates of medical reviews sought following vaccination are used for safety signal detection [8] in conjunction with other data, such as from the TGA’s national spontaneous AEFI reporting system [9].

While the short-term post-licensure safety profile of Spikevax has been thoroughly described through active surveillance [10,11,12], less evidence exists for Nuvaxovid [13]. In Australia, the short-term safety of Spikevax and Nuvaxovid was reported in a 2022 study [14]; however, the study only included people who had received vaccination through pharmacies, and in the case of Nuvaxovid, just 1527 day 3 safety surveys were completed across dose 1, dose 2, dose 3, and booster doses. To further define the safety profile of COVID-19 vaccine priming doses in the Australian context, analysis of reported AEFI in a larger cohort across a variety of immunisation providers and with more granularity is warranted.

This study describes the short-term adverse event profile of the priming doses of Spikevax and Nuvaxovid vaccines as measured by AusVaxSafety.

## 2. Materials and Methods

People aged 12 years and above who received the Spikevax or Nuvaxovid vaccine as a primary course (dose 1 and dose 2) at an AusVaxSafety sentinel surveillance site were eligible for inclusion in this study. Vaccine encounters that occurred between September 2021 and September 2023 were included in the study.

Enrolment to receive the surveys was either opt in or automated enrolment (i.e., opt out), depending on logistical factors by location across the eight Australian states and territories: people who received their vaccination through a New South Wales, Victorian, or Western Australian Government vaccination hub, participating general practices, and pharmacies or through a Western Australian Aboriginal Community Controlled Health Organisation (ACCHO) were automatically enrolled to receive the surveys but could opt out of completion; all other Government vaccination hubs (Queensland, Australian Capital Territory, Northern Territory, South Australia, and Tasmania) and all other ACCHOs employed a quick response (QR) code enrolment system allowing vaccine recipients to opt in to enrolment to receive the surveys.

Surveys were delivered via AusVaxSafety linked surveillance tools: Vaxtracker [15], SmartVax [16], and the COVID-19 Vaccination Management System (CVMS) [17,18]. Links to the web-based survey were sent via SMS or email on the third and eighth day after vaccination and asked for responses based on days 0–3 (day 3 survey) and days 4–7 (day 8 survey) after vaccination. Day 8 surveys were only sent to respondents of day 3 surveys and asked about new or ongoing symptoms. Due to an AusVaxSafety policy change, day 8 surveys ceased being sent on 3 July 2023. The surveys included categorical questions on solicited adverse events (local reactions, subjective fever, rash, chills, headaches, myalgia, arthralgia, gastrointestinal symptoms, fatigue, loss of consciousness, or seizure) and unsolicited adverse events (free text), whether medical review was sought (phone advice, general practitioner or Aboriginal healthcare worker, or emergency department), impact of adverse event(s) on usual activities, symptom resolution, pre-existing medical conditions, and pregnancy (see Deng et. al. [1] for survey questions). A reminder SMS or email was sent the next day if a response had not been received. Only responses received by day 7 after vaccination for the day 3 survey and day 14 for the day 8 survey were included for analysis to reduce the risk of errors in recall. Where day 8 data are reported, only respondents who provided a response for both the day 3 and day 8 surveys were included.

All analyses were conducted in R (R Foundation for Statistical Computing) [19] version 4.3.1 for descriptive analyses and version 4.1.3 for Bayesian analyses. Participant demographic information (date of birth, sex, Indigenous status) and vaccination details (vaccine brand, dose, batch number, vaccination date) were obtained via the surveillance tools from vaccination records or from self-registration. Survey participants who answered ‘yes’ to the question ‘Did you experience any other symptoms not listed above?’ were able to describe their symptoms in a free-text field. A regular expression search string was used to extract chest pain/discomfort symptoms from the free-text field. The search string used MedDRA^®^ (the Medical Dictionary for Regulatory Activities) version 26.0 [20] lower-level terms (LLTs) that mapped to MedDRA preferred terms (PTs) of “chest pain” and “chest discomfort” (see Supplementary Materials for the search string used).

Descriptive statistics were used to describe the demographic characteristics of the sample as well as the percentage of respondents who reported any adverse event, solicited adverse events or chest pain/discomfort symptoms, medical review, impact on usual activities, and symptom resolution.

The proportion of participants who reported AEFI in the day 3 survey for each vaccine brand and dose number was modelled using Bayesian logistic regression, similar to the analysis described in Deng et al. [1]. An interaction term was modelled for age and sex to account for sex-specific age effects, as suggested by evidence presented in Table S3, Supplementary Materials. The model also adjusted for Indigenous status, clinic type, jurisdiction, anaphylaxis history, and underlying medical condition. Survey responses reporting sex as other (*n* = 47), missing age (*n* = 40), or missing Indigenous status (*n* = 1136) were excluded from analysis (2.3%). To address the 9204 day 3 survey responses (18.1%) with missing sex data, the majority of which were self-reports collected from pharmacy sites, the model was marginalised over sex for these respondees (i.e., assumed that sex was missing at random) [21,22]. Posterior distributions for model parameters were estimated using STAN [20] via the *cmdstanr* R package (version 0.6.1) [21]. Each model was run with 8 chains of 1000 iterations, and model convergence was assessed using *cmdstanr* diagnostic tools (see Supplementary Materials for further information).

## 3. Results

### 3.1. Sample Characteristics

A total of 131,775 day 3 post-vaccination surveys and 43,875 day 8 surveys, matched to responders for the day 3 surveys, were sent to people who received either a Spikevax or Nuvaxovid primary dose at an AusVaxSafety sentinel site. We received 50,721 day 3 survey responses (overall response rate, 38.5%; Spikevax dose 1, 42.3%; dose 2, 34.0%; Nuvaxovid dose 1, 41.5%; dose 2: 39.5%) and 31,355 day 8 survey responses (overall response rate, 71.5%; Spikevax dose 1, 71.7%; dose 2, 72.3%; Nuvaxovid dose 1, 66.3%; dose 2: 69.4%). See Table S2 in the Supplementary Materials for full details of the response rates. The median age of respondents was higher for Nuvaxovid than Spikevax for both doses. More females than males completed the survey for each vaccine brand/vaccine dose/survey day combination; however, sex was missing for 14,949 day 3 and day 8 surveys (18.2%), predominantly from the pharmacy sites. Aboriginal and Torres Strait Islander people (hereafter respectfully referred to as Aboriginal) represented 2.9% of Spikevax respondents for the day 3 survey and 2.8% for the day 8 survey and 2.1% of Nuvaxovid respondents for the day 3 and 2.2% for the day 8 survey. See Table S3 in the Supplementary Materials for respondent demographic characteristics.

### 3.2. Adverse Events 0–3 and 4–7 Days after Vaccination

**Figure 1:**
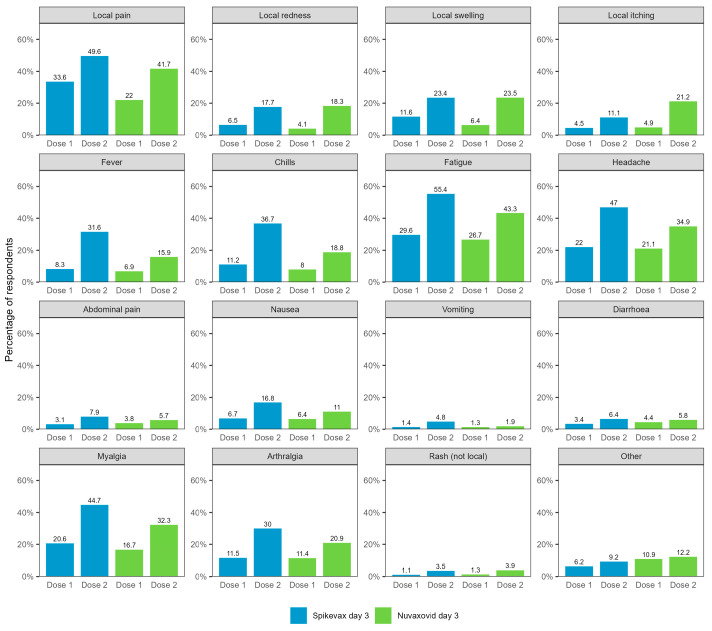
Reported adverse events 0–3 days after vaccination with Spikevax or Nuvaxovid for all day 3 respondents (*N* = 50,721). Not shown in figure: self-reported fainting/loss of consciousness (Spikevax dose 1: 0.84%, dose 2: 1.51%; Nuvaxovid dose 1: 0.81%, dose 2: 1.00%) and possible seizure (Spikevax dose 1: 0.09%, dose 2: 0.15%; Nuvaxovid dose 1: 0.07%, dose 2: 0.21%).

Of the total 50,721 day 3 survey responses received, half of respondents (50.7%) reported any adverse event in the three days following immunisation (Spikevax dose 1: 41.7%, dose 2: 64.2%; Nuvaxovid dose 1: 36.6%, dose 2: 57.3%) (Table 1). Rates of reported adverse events were higher for dose 2 than dose 1 for both vaccines. The most commonly reported adverse events 0–3 days after vaccination included fatigue, pain at the injection site, headaches, myalgia, and arthralgia (Figure 1).

**Figure 2:**
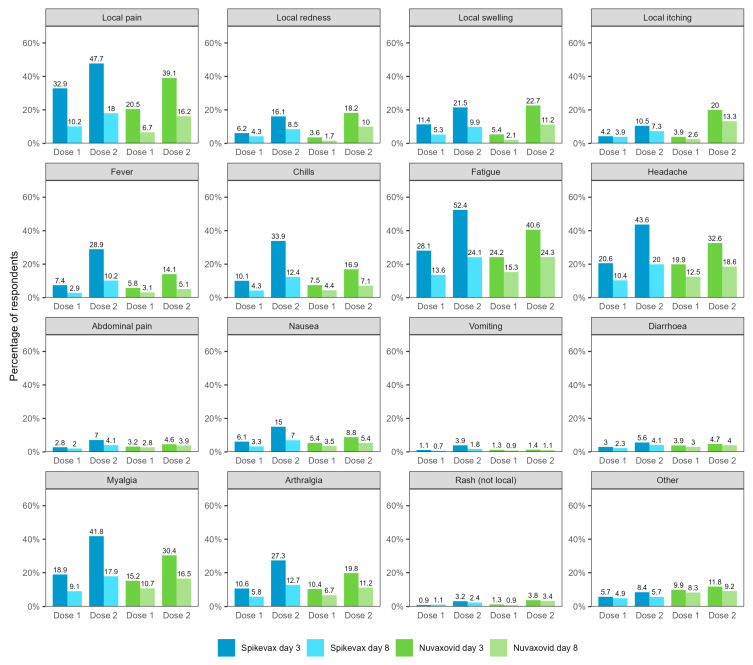
Reported adverse events 0–3 and 4–7 days after vaccination with Spikevax or Nuvaxovid for respondents who completed both the day 3 and the day 8 survey (*N* = 31,355). Not shown in figure: self-reported fainting/loss of consciousness (Spikevax dose 1/day 3: 0.61%, Spikevax dose 1/day 8: 0.31%, Spikevax dose 2/day 3: 1.07%, Spikevax dose 2/day 8: 0.54%; Nuvaxovid dose 1/day 3: 0.79%, Nuvaxovid dose 1/day 8: 0.37%, Nuvaxovid dose 2/day 3: 0.96%, Nuvaxovid dose 2/day 8: 0.64%) and possible seizure (Spikevax dose 1/day 3: 0.06%, Spikevax dose 1/day 8: 0.04%, Spikevax dose 2/day 3: 0.08%, Spikevax dose 2/day 8: 0.10%; Nuvaxovid dose 1/day 3: 0.10%, Nuvaxovid dose 1/day 8: 0.05%, Nuvaxovid dose 2/day 3: 0.24%, Nuvaxovid dose 2/day 8: 0.16%).

Figure 2 compares reported adverse events between the day 3 and day 8 surveys of respondents who completed both surveys (*N* = 31,355). In this cohort, 48.8% reported any adverse event 0–3 days after vaccination (Spikevax dose 1: 40.5%, dose 2: 61.7%; Nuvaxovid dose 1: 34.3%, dose 2: 54.9%) and 24.6% reported any adverse event 4–7 days after vaccination (Spikevax dose 1: 20.2%, dose 2: 30.1%; Nuvaxovid dose 1: 22.1%, dose 2: 34.1%). The frequency of all adverse events was lower in the day 8 surveys except for rash following dose 1 of Spikevax (day 3: 0.9%, day 8: 1.1%).

Reported chest pain/discomfort rates were similar between vaccines in both the day 3 survey (Spikevax dose 1: 0.6%, dose 2: 0.8%; Nuvaxovid dose 1: 1.0%, dose 2: 1.3%) and the day 8 survey (Spikevax dose 1: 0.4%, dose 2: 0.5%; Nuvaxovid dose 1: 1.1%, dose 2: 1.1%).

**Figure 3:**
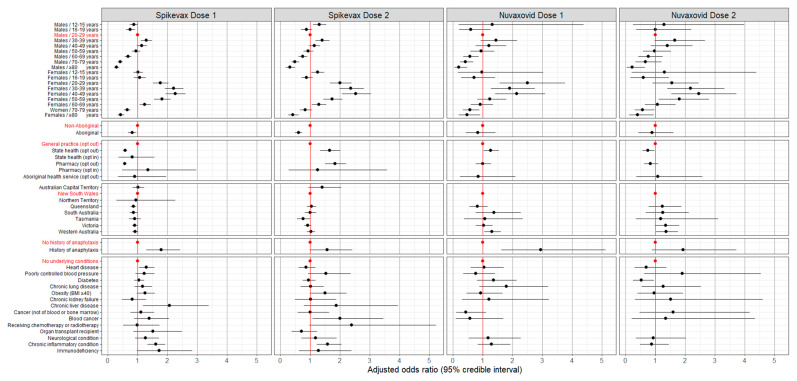
Any adverse event 0–3 days after vaccination with Spikevax or Nuvaxovid: adjusted odds ratios by vaccine, dose, and respondent characteristics (*N* = 48,178). Reference levels for each categorical variable are indicated in red. Odds ratios are adjusted for demographic characteristics, history of anaphylaxis, and underlying medical conditions and for clinic type and jurisdiction. The full data for this graph are included in the Supplementary Materials, Table S4.

**Figure 4:**
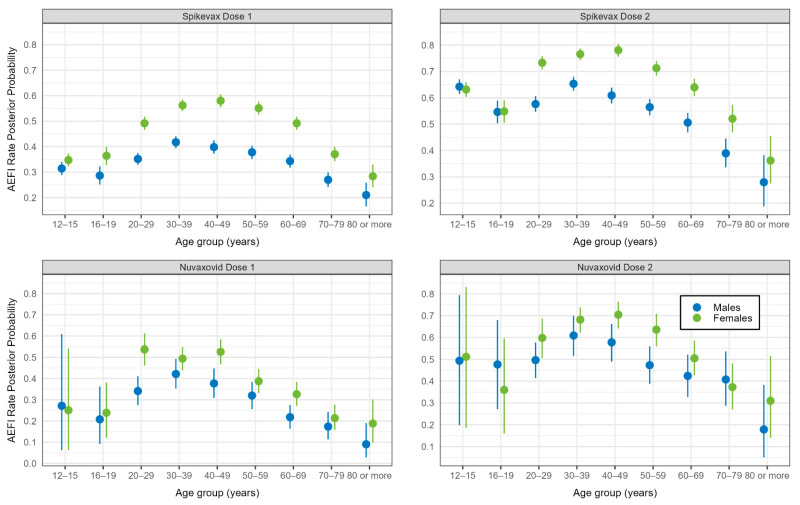
Any adverse event 0–3 days after vaccination with Spikevax or Nuvaxovid: mean posterior probability (with 95% credible intervals), by dose, age group, and sex (*N* = 48,178).

There was no evidence of lack of convergence in any of the Bayesian models. After adjusting for clinic type, jurisdiction, medical conditions, and demographic characteristics, the odds of reporting AEFI rose with increasing age from 16–19 years of age to 30–39 years, after which there was a decline in the odds with increasing age for both vaccines and doses. The effect of age on the odds of reporting AEFI was greater in females and less pronounced from the 50–59-year age group onwards in both vaccine brands and doses, but this effect was less noticeable with Novavax. The effect of sex was not apparent in the 12–15-year and 16–19-year age groups. (Figure 3 and Figure 4).

Aboriginal respondents were less likely to report AEFI following Spikevax (dose 1: adjusted odds ratio (aOR), 0.79; 95% credible interval (CrI), 0.67–0.92; dose 2: aOR, 0.59, 95% CrI, 0.49–0.70) compared to non-Aboriginal respondents. Respondents with a history of anaphylaxis were more likely to report any AEFI than those who did not report a history of anaphylaxis, with the effect greater for Nuvavoxid (dose 1: aOR, 2.86; 95% CrI, 1.56–4.85; dose 2: aOR, 1.72; 95% CrI, 0.81–3.26) compared to Spikevax (dose 1: aOR, 1.76; 95% CrI, 1.27–2.37; dose 2: aOR, 1.50; 95% CrI, 0.97–2.26) (Figure 3). Just over half (59%) of the respondents with a history of anaphylaxis reported any AEFI, with 18.7% of these respondents seeking medical attention from a GP or Aboriginal healthcare worker and 3.6% of these respondents stating they attended ED for the AEFI.

Due to the wide credible intervals, indicating low precision, there is no consistent evidence of any difference in the odds of AEFI for many of the reported underlying medical conditions compared to no underlying conditions (Figure 3). See Table S4 in the Supplementary Materials for full details of the aORs.

### 3.3. Impact on Routine Activities and Medical Review

Impact on routine activities ranged from 13.3% to 35.0% after vaccination with Spikevax and 12.9% to 25.0% after Nuvaxovid (Table 2). More than half of respondents who completed both day 3 and day 8 surveys, and reported an AEFI in the day 3 survey, reported that their symptoms had resolved by day 3 after vaccination with Spikevax (dose 1: 67.7% and dose 2: 61.1%); this figure was just under half after Nuvaxovid (dose 1: 47.6% and dose 2: 45.0%). By day 8, these figures increased for all vaccine brand/dose combinations. A total of 1578 (3.1%) respondents reported requiring medical review following vaccination, ranging from 1.9% to 4.8% after vaccination with Spikevax and 2.7% to 3.9% after Nuvaxovid. Overall, 326 respondents (0.6%) reported visiting an emergency department following vaccination.

## 4. Discussion

We report AEFI experienced in the week following vaccination with Spikevax or Nuvaxovid, extending the available data on the short-term safety profile of priming doses provided in Australia. AEFI were reported more frequently among people vaccinated with Spikevax than Nuvaxovid for both dose 1 and dose 2, with the highest proportion of recipients reporting AEFI following dose 2 of Spikevax. This was consistent with a study by Salter et al., which included respondents from Australian pharmacies only and who make up part of the cohort in this study [14].

The self-reported rates of AEFI following Spikevax and Nuvaxovid vaccinations (Spikevax dose 1: 41.7%, dose 2: 64.2%; Nuvaxovid dose 1: 36.6%, dose 2: 57.3%) were notably lower than those observed in phase 3 clinical trials [23,24]. This is likely due to a more rigorous method of collecting AEFI reports via daily solicitation in clinical trials. Rates of reported AEFI after vaccination with Spikevax were also lower in this study compared to international active safety surveillance in the United States [10], the Netherlands [11], and Spain [12] and may be due to variation in methodologies; the United States study, for example, uses an opt in only approach. However, AEFI rates for Nuvaxovid in this study were comparable to those described by Kim et al. in the Republic of Korea [13]. Whilst the absolute rates of AEFI were lower in our study, the pattern of local and systemic adverse events reported align with clinical trials and post-licensure surveillance [10,11,12,13,23,24].

Adjusted odds ratio analysis showed that individuals aged 30–39 years had the highest likelihood of reporting AEFI in the three days following vaccination across all vaccine brand/dose combinations. Consistent with other studies [1,10,11,12,13,14,23,24] a decline in the odds of AEFI reporting is noted with increasing age beyond the 30–39-year age group. Overall, females had greater odds of reporting AEFI compared with males across most age bands, vaccine types, and doses observed in the Deng et al. study [1]. Aboriginal respondents had lower odds of reporting AEFI in the three days following vaccination with Spikevax than non-Aboriginal respondents. The cohort in this study had a higher proportion of Aboriginal respondents (2.7%) compared to the Deng et al. study (1.3%) and is more similar to the Australian population proportion of 3.8% [25]. AusVaxSafety sentinel sites include ACCHOs in most states and territories; however, increased recruitment of ACCHOs is warranted to assist with capturing a representative sample of Aboriginal people across the country.

Medical review was reported by 3.1% of respondents, with the highest rate occurring after the second dose of each vaccine (Spikevax dose 2: 4.8%, Nuvaxovid dose 2: 3.9%). Additionally, 0.6% of all respondents reported visiting an emergency department (ED), which is higher than the rates observed in those who received Comirnaty and Vaxzevria vaccines in the initial six months of the COVID-19 vaccine rollout (0.9% medical review and 0.2% ED presentations) [1]. This cohort also reported a greater impact of vaccination on usual activities, with a higher proportion reporting missing two days (range: 29.5% to 34.5%) or three or more days (range: 14.3 to 24.4%) of usual activity compared to the cohort who were vaccinated with Comirnaty or Vaxzeveria (range for two days: 22.4% to 24.6%, range for three or more days: 5.4% to 6.6%). The disparities in medical review and impact on usual activity may stem from a different (and potentially more vaccine-hesitant) cohort more inclined to seek medical review for common expected AEFIs. Nuvaxovid was approved for use in January 2022 when 92.8% of the eligible population had already completed their COVID-19 priming doses [26], and it is hypothesised that some people who were hesitant to receive an mRNA vaccine due to the reported association with myo-\peri\carditis may have delayed vaccination in order to receive Nuvaxovid [27]. Notably, the self-reported rate of chest pain/discomfort in our study was comparable between vaccines and ranged from 0.6% to 1.3%. Other considerations are the changes in the pandemic situation and control measures [28] since the Deng et al. study. However, it is important to note that reported adverse events in this study have not been further clinically investigated, and causality from vaccination cannot be inferred due to the temporal relationship alone; some AEFI may be linked to other causes, such as intercurrent infection with circulating viruses (resulting in medical review or time off normal duties) rather than being associated with the vaccine. Nevertheless, reported AEFI appear transient, with specific AEFI rates lower in the day 8 survey compared to the day 3 survey.

Several limitations merit consideration in interpreting our study’s results. As previously described in Deng et al. [1], AEFI rates reported through the surveys do not provide direct evidence of causation, and the true rate of AEFI is likely to be overestimated if people who experience an AEFI are more likely to respond to the survey than those who do not. People from culturally and linguistically diverse populations may be underrepresented, as the survey is provided in English only, and people who do not have access to a smartphone or the internet would not be able to participate. The sample size was much smaller for Nuvaxovid (4856 day 3 surveys), likely due to being introduced later in the vaccine rollout, combined with a lack of vaccine availability, compared to Spikevax (45,865 day 3 surveys), providing less precision on adjusted odds ratios. Additionally, using a regular expression search string to extract chest pain/discomfort symptoms from the free text field was a less sensitive approach to detect all relevant free text responses, compared to assessing each response manually. Finally, 18.1% of day 3 surveys had missing data for sex of survey participants. However, this was likely missing at random, and Bayesian methodology marginalising the missing sex data was employed to retain data relating to other covariates for the analysis.

## 5. Conclusions

Active vaccine safety surveillance systems, like AusVaxSafety, complement passive AEFI surveillance methods, offering a comprehensive dataset on vaccine safety for both providers and consumers in the post-licensing phase. Our findings, along with Deng et al., affirm the short-term reactogenicity but also expected good safety profile of all four COVID-19 vaccines administered as primary courses in the Australian population. AusVaxSafety remains vigilant in monitoring the safety of COVID-19 vaccines in Australia.

## Figures and Tables

**Table 1:** Rates of any adverse event following COVID-19 vaccination, as reported in the AusVaxSafety day 3 COVID-19 vaccine safety survey by vaccine, dose number, and selected respondent characteristics (*N* = 50,721).

	Spikevax		Nuvaxovid	
Characteristic	Dose 1 *n*/*N* (%)	Dose 2 *n*/*N* (%)	Dose 1 *n*/*N* (%)	Dose 2 *n*/*N* (%)
All respondents	10,948/26,254 (41.7%)	12,585/19,611 (64.2%)	1088/2972 (36.6%)	1079/1884 (57.3%)
Sex ^1^				
Female	5553/11,615 (47.8%)	5394/7817 (69.0%)	624/1518 (41.1%)	579/946 (61.2%)
Male	3732/10,557 (35.4%)	4263/7314 (58.3%)	335/1084 (30.9%)	313/619 (50.6%)
Other	18/30 (60.0%)	12/15 (80.0%)	<5 (100.0%)	<5 (100.0%)
Missing data	4052	4465	369	318
Age (years) ^1^				
Median (IQR)	39 (26, 55)	35 (21, 49)	43 (33, 56)	43 (34, 55)
Missing data	13	9	10	8
Indigenous status ^1^				
Aboriginal	276/733 (37.7%)	287/529 (54.3%)	21/61 (34.4%)	20/41 (48.8%)
Non-Aboriginal	10,405/24,907 (41.8%)	12,028/18,652 (64.5%)	1037/2858 (36.3%)	1031/1804 (57.2%)
Missing data	614	430	53	39
Anaphylaxis history				
No	10,618/25,634 (41.4%)	12,242/19,145 (63.9%)	1008/2833 (35.6%)	1007/1781 (56.5%)
Yes	330/620 (53.2%)	343/466 (73.6%)	80/139 (57.6%)	72/103 (69.9%)
Underlying medical condition				
No	9046/22,634 (40.0%)	11,129/17,510 (63.6%)	834/2374 (35.1%)	896/1566 (57.2%)
Yes	1902/3620 (52.5%)	1456/2101 (69.3%)	254/598 (42.5%)	183/318 (57.5%)

^1^ Missing data not included in descriptive analyses. IQR = interquartile range. *n*/*N* = *n* represents the number of respondents who reported any adverse event within that population/vaccine brand/vaccine dose subgroup and *N* represents the total number of people who responded to the day 3 survey within that population/vaccine brand/vaccine dose subgroup.

**Table 2:** Impact on daily activities, medical review, and symptom resolution 0–3 days after vaccination by vaccine and dose (*N* = 50,721).

	Spikevax		Nuvaxovid	
Characteristic	Dose 1 *n*/*N* (%)	Dose 2 *n*/*N* (%)	Dose 1 *n*/*N* (%)	Dose 2 *n*/*N* (%)
Missed work, study, or routine activities ^1^	*N* = 26,254	*N* = 19,611	*N* = 2972	*N* = 1884
	3493/26,220 (13.3%)	6849/19,583 (35.0%)	383/2971 (12.9%)	471/1882 (25.0%)
Missing	34	28	1	2
Number of days missed from routine activities ^1^	*N* = 3493	*N* = 6849	*N* = 383	*N* = 471
Less than a day	326/3484 (9.4%)	470/6836 (6.9%)	35/378 (9.3%)	30/471 (6.4%)
One day	1462/3484 (42.0%)	3032/6836 (44.4%)	126/378 (33.3%)	187/471 (39.7%)
Two days	1179/3484 (33.8%)	2358/6836 (34.5%)	130/378 (34.4%)	139/471 (29.5%)
Three or more days	517/3484 (14.8%)	976/6836 (14.3%)	87/378 (23.0%)	115/471 (24.4%)
Missing data	9	13	5	0
Medical review sought	*N* = 26,254	*N* = 19,611	*N* = 2972	*N* = 1884
	493/26,254 (1.9%)	931/19,611 (4.8%)	81/2972 (2.7%)	73/1884 (3.9%)
Highest level medical review obtained ^1^	*N* = 493	*N* = 931	*N* = 81	*N* = 73
ED	113/450 (25.1%)	182/869 (20.9%)	20/75 (26.7%)	11/72 (15.3%)
GP or Aboriginal HCW	201/450 (44.7%)	409/869 (47.1%)	39/75 (52.0%)	40/72 (55.6%)
Phone	136/450 (30.2%)	278/869 (32.0%)	16/75 (21.3%)	21/72 (29.2%)
Missing data	43	62	6	1
Symptom resolution by day 3 ^1,2^	*N* = 6585	*N* = 7362	*N* = 655	*N* = 683
	4443/6559 (67.7%)	4479/7334 (61.1%)	311/653 (47.6%)	306/680 (45.0%)
Missing data	26	28	2	3
Symptom resolution by day 8 ^1,2^	*N* = 6585	*N* = 7362	*N* = 655	*N* = 683
	4800/6499 (73.9%)	5385/6708 (80.3%)	357/629 (56.8%)	422/633 (66.7%)
Missing data	86	654	26	50

^1^ Missing data not included in descriptive analyses. ^2^ Denominator is number of respondents who completed both day 3 and day 8 surveys and reported at least one adverse event in the day 3 survey (*N* = 15,285). *n*/*N* = *n* represents the number of respondents who reported the specified characteristic within that vaccine brand/vaccine dose subgroup and *N* represents the total number of people who responded to that characteristic in the day 3 survey within that vaccine brand/vaccine dose subgroup.

## Data Availability

The datasets presented in this article are not readily available because the data are owned by the Australian Government.

## Supplementary Materials

The following supporting information can be downloaded at: https://www.mdpi.com/article/10.3390/vaccines12090971/s1, Table S1: Notation of covariate levels for the age group and sex interaction terms in responders with reported sex (a), the age group terms in responders with missing sex (b) and additional covariate terms in all responders (c); Table S2: Response rates of day 3 and day 8 survey respondents to the AusVaxSafety COVID-19 vaccine safety surveys, by vaccine and dose number; Table S3: Demographic characteristics of day 3 and day 8 survey respondents to the AusVaxSafety COVID-19 vaccine safety surveys, by vaccine and dose number; Table S4: Any adverse event following COVID-19 vaccination, as reported in the AusVaxSafety day 3 COVID-19 vaccine safety survey, September 2021 to September 2023, by vaccine, dose number, and respondent characteristics.
