# Supplementary material for: Short-Term Active Safety Surveillance of the Spikevax and Nuvaxovid Priming Doses in Australia
Source: Vaccines (Basel). 2024 Aug 27;12(9):971. doi: 10.3390/vaccines12090971 (PMC11435866; doi:10.3390/vaccines12090971)
Supplement: Supplementary file 1 [file vaccines-12-00971-s001.zip › vaccines-3139046-supplementary.pdf]

1. Regular expression search string to extract chest pain/discomfort symptoms

"\\b((chest|substern*|precordial|retrostern*|parastern*|thoracic|thorax|sternal).*(pain*|burn*|ach*|distress)|(pain*|burn*|ach*|distress).*(chest|substern*|precordial|retrostern*|parastern*|thoracic|thorax|sternal))\\b"

2. Bayesian logistic regression model

Bayesian logistic regression models were used to model the proportion of participants reporting any AEFI in the Day 3 survey for each vaccine brand and dose number. The model adjusts for responder age group and sex (interaction), Indigenous status, clinic type, jurisdiction, anaphylaxis history and underlying medical condition. Responses with sex reported as other or missing age or Indigenous status were excluded. A high proportion of responses were missing sex data (18.1%) and so the model marginalises over sex for these responses. Here, marginalisation assumes that sex was missing at random and that the proportion of males and females responding to the survey in this group (those with missing sex) was the same as in the responders that reported sex. The reference levels for the covariates age group and sex interaction, Indigenous status, clinic type, jurisdiction, anaphylaxis history and underlying medical condition were 20-29 year old males, non-Aboriginal, general practice – opt out, NSW, no history of anaphylaxis and no underlying medical condition, respectively.

Denote responders as $i \in I_s \cup I_{\bar{s}}$, where $I_s \cap I_{\bar{s}} = \emptyset$. Here, responders belong to the non-overlapping sets I_s and $I_{\bar{s}}$ if they have reported sex or are missing sex, respectively. The sex and outcome for responder i are denoted $s_i, y_i \in \{0,1\}$, respectively (note that s_i is defined only for $i \in I_s$).

We separate out the covariates into three vectors: $\boldsymbol{z_i}$, $\boldsymbol{w_i}$ and $\boldsymbol{x_i}$. Let $\boldsymbol{z_i}$ contain the age group and sex interaction terms for responders that reported sex and $\boldsymbol{w_i}$ contain the age group terms for responders with missing sex. Here, $\boldsymbol{z_i}$ and $\boldsymbol{w_i}$ are vectors of length 17 (9 age groups times 2 sex levels minus 1 reference level) and 8 (9 age groups minus 1 reference level), respectively, with details provided in Tables 1a and 1b. We denote an additional variable w_i^* to indicate responder i with missing sex belonging to the reference age group (20-29 years). Let $\boldsymbol{x_i}$ be of length 29 and contain the covariate terms for Indigenous status, clinic type, jurisdiction, anaphylaxis history and underlying medical condition with details provided in Table 1c.

The likelihood for responder sex in responders with reported sex is:

$$s_i \sim \text{Bernoulli}(p) \;\; \forall i \in I_s$$

$$\text{logit}(p) = \delta$$

Here, p is the proportion of female responders in the population of responders. The likelihood for reported AEFI in responders with reported sex is:

$$y_i \sim \text{Bernoulli}\left(\text{logit}^{-1}\left(f_s(i)\right)\right) \;\; \forall i \in I_s$$

$$f_s(i) = \alpha + \sum_{q=1}^{17} z_{iq}\gamma_q + \sum_{k=1}^{29} x_{ik}\beta_k$$

Here, α is the log odds of a responder belonging to each of the covariate reference levels reporting AEFI, γ_q is the log odds ratio for age group and sex interaction term q and β_k is the log odds ratio for covariate term k.

The likelihood for reported AEFI in responders with missing sex is:

$$y_i \sim \text{Bernoulli}\left(p \times \text{logit}^{-1}\left(f_f(i)\right) + (1-p) \times \text{logit}^{-1}\left(f_m(i)\right)\right) \quad \forall i \in I_{\bar{s}}$$

$$f_f(i) = \alpha + w_i^*\gamma_{11} + \sum_{q=1}^{8} w_{iq}\gamma_q^f + \sum_{k=1}^{29} x_{ik}\beta_k$$

$$f_m(i) = \alpha + \sum_{q=1}^{8} w_{iq}\gamma_q^m + \sum_{k=1}^{29} x_{ik}\beta_k$$

Where,

$$\boldsymbol{\gamma^f} = \boldsymbol{M_f\gamma}$$

$$\boldsymbol{\gamma^m} = \boldsymbol{M_m\gamma}$$

Here, we marginalise over sex by averaging the inverse logit transformed linear predictors for females (f_f) and males (f_m) by the estimated proportion of females (p) and males ($1-p$) in the responder population. The sex-specific linear predictors only differ by the inclusion of the sex-specific age group and sex interaction terms, controlled by the matrices $\boldsymbol{M_f}$ and $\boldsymbol{M_m}$, which map the appropriate age group and sex interaction terms from the parameter vector $\boldsymbol{\gamma}$ to the sex-specific parameter vectors $\boldsymbol{\gamma^f}$ and $\boldsymbol{\gamma^m}$ for females and males, respectively. For the female-specific linear predictor, we also include the term γ_{11} (corresponding to the 20-29 years / female age group and sex interaction term) if and only if responder i is in the reference age group (20-29 years).

The weakly informative prior distributions are:

$$\delta \sim \text{Normal}(0,1)$$

$$\alpha \sim \text{Normal}(0,1)$$

$$\gamma_q \sim \text{Normal}(0,1)\ \forall q \in \{1,2,\dots,17\}$$

$$\beta_k \sim \text{Normal}(0,1)\ \forall k \in \{1,2,\dots,29\}$$

The model was implemented in STAN via the following code (note that subscripts *obs* and *miss* are used to denote responders that have reported sex and missing sex, respectively):

```
data{
  int N_obs;                              // number of responses where sex is observed
  int N_miss;                             // number of responses where sex is missing
  array[N_obs] int sex_obs;               // observed sex (male == 0, female == 1)
  int N_z;                                // number of age group x sex interaction terms
  int N_w;                                // number of age group terms
  int N_x;                                // number of additional covariate terms
  matrix[N_obs, N_z] Z;                   // covariate matrix for age group x sex interaction terms
  matrix[N_miss, N_w] W;                  // covariate matrix for age group terms
  matrix[N_obs, N_x] X_obs;               // covariate matrix for those with observed sex
  matrix[N_miss, N_x] X_miss;             // covariate matrix for those with missing sex
  array[N_miss] int w_star;               // indicator for reference age group for responses where sex is missing
  matrix[N_w, N_z] M_f;                   // matrix to map female-specific gammas
  matrix[N_w, N_z] M_m;                   // matrix to map male-specific gammas
  array[N_obs] int y_obs;                 // outcomes for those with observed sex
  array[N_miss] int y_miss;               // outcomes for those with missing sex
}

parameters{
  real delta;                             // population proportion of female (logit transformed)
  real alpha;                             // intercept term
  vector[N_z] gamma;                      // parameter vector for age group x sex interaction terms
  vector[N_x] beta;                       // parameter vector for additional covariate terms
}

transformed parameters{
  vector[N_w] gamma_f = M_f*gamma;      // extracted gamma parameters corresponding to females
  vector[N_w] gamma_m = M_m*gamma;  // extracted gamma parameters corresponding to males
}

model{
  // weakly informative prior distributions (note: alpha is on the log-odds scale)

  target += std_normal_lpdf(delta);
  target += std_normal_lpdf(alpha);
  target += std_normal_lpdf(gamma);
  target += std_normal_lpdf(beta);

  // distribution for sex

  target += bernoulli_logit_lpmf(sex_obs | delta);

  // observed component

  target += bernoulli_logit_lpmf(y_obs | alpha + Z*gamma + X_obs*beta);

  // missing component (note: w_star[i]*gamma[11] is included to account for females aged 20-29 that would
otherwise be missed)

  for(i in 1:N_miss)
    target += log_mix(inv_logit(delta),
                         bernoulli_logit_lpmf(y_miss[i] | alpha + w_star[i]*gamma[11] + W[i,]*gamma_f +
X_miss[i,]*beta),
                        bernoulli_logit_lpmf(y_miss[i] | alpha +                         W[i,]*gamma_m +
X_miss[i,]*beta));
}
```

Table S1. Notation of covariate levels for the age group and sex interaction terms in responders with reported sex (a), the age group terms in responders with missing sex (b) and additional covariate terms in all responders (c).

a

Notation	Age Group	Sex
z_{i1}	12-15 years	Male
z_{i2}	16-19 years	Male
z_{i3}	30-39 years	Male
z_{i4}	40-49 years	Male
z_{i5}	50-59 years	Male
z_{i6}	60-69 years	Male
z_{i7}	70-79 years	Male
z_{i8}	≥80 years	Male
z_{i9}	12-15 years	Female
z_{i10}	16-19 years	Female
z_{i11}	20-29 years	Female
z_{i12}	30-39 years	Female
z_{i13}	40-49 years	Female
z_{i14}	50-59 years	Female
z_{i15}	60-69 years	Female
z_{i16}	70-79 years	Female
z_{i17}	≥80 years	Female

b

Notation	Age Group
w_i^*	20-29 years
w_{i1}	12-15 years
w_{i2}	16-19 years
w_{i3}	30-39 years
w_{i4}	40-49 years
w_{i5}	50-59 years
w_{i6}	60-69 years
w_{i7}	70-79 years
w_{i8}	≥80 years

c

Notation	Level	Covariate
x_{i1}	Aboriginal	Indigenous Status
x_{i2}	State Health – Opt out	Clinic Type
x_{i3}	State Health – Opt in	Clinic Type
x_{i4}	Pharmacy – Opt out	Clinic Type
x_{i5}	Pharmacy – Opt in	Clinic Type
x_{i6}	Aboriginal Health Service – Opt out	Clinic Type
x_{i7}	ACT	Jurisdiction
x_{i8}	NT	Jurisdiction
x_{i9}	QLD	Jurisdiction
x_{i10}	SA	Jurisdiction
x_{i11}	TAS	Jurisdiction
x_{i12}	VIC	Jurisdiction
x_{i13}	WA	Jurisdiction
x_{i14}	History of Anaphylaxis	Anaphylaxis History
x_{i15}	Heart Disease	Underlying Medical Condition
x_{i16}	High blood pressure	Underlying Medical Condition
x_{i17}	Diabetes	Underlying Medical Condition
x_{i18}	Lung Disease	Underlying Medical Condition
x_{i19}	Obesity	Underlying Medical Condition
x_{i20}	Kidney Disease	Underlying Medical Condition
x_{i21}	Liver Disease	Underlying Medical Condition
x_{i22}	Cancer	Underlying Medical Condition
x_{i23}	Blood cancer	Underlying Medical Condition
x_{i24}	Chemotherapy or radiation	Underlying Medical Condition
x_{i25}	Organ transplant	Underlying Medical Condition
x_{i26}	Bone marrow transplant	Underlying Medical Condition
x_{i27}	Neurological condition	Underlying Medical Condition
x_{i28}	Inflammatory condition	Underlying Medical Condition
x_{i29}	Immunodeficiency	Underlying Medical Condition

Table S2. Response rates of day 3 and day 8 survey respondents to the AusVaxSafety COVID-19 vaccine safety surveys, by vaccine and dose number*

	Spikevax		Nuvaxovid		
**Characteristic**	**Dose 1**	**Dose 2**	**Dose 1**	**Dose 2**	
**Day 3 survey**					**Total**
Number of respondents	26,254	19,611	2,972	1,884	50,721
Number of surveys sent	62,094	57,743	7,163	4,775	131,775
Response rate (%)	42.3	34.0	41.5	39.5	38.5
**Day 8 survey**					**Total**
Number of respondents	16,264	11,937	1,909	1,245	31,355
Number of surveys sent	22,697	16,505	2,880	1,793	43,875
Response rate (%)	71.7	72.3	66.3	69.4	71.5

*Only day 8 surveys where the respondent had also answered a day 3 survey were included.

Table S3. Demographic characteristics of day 3 and day 8 survey respondents to the AusVaxSafety COVID-19 vaccine safety surveys, by vaccine and dose number*

	Spikevax				Nuvaxovid			
**Characteristic[a]**	**Dose 1**		**Dose 2**		**Dose 1**		**Dose 2**	
	**Day 3 survey**	**Day 8 survey**	**Day 3 survey**	**Day 8 survey**	**Day 3 survey**	**Day 8 survey**	**Day 3 survey**	**Day 8 survey**
Number of respondents	26,254	16,264	19,611	11,937	2,972	1,909	1,884	1,245
Sex, n (%)								
Female	11,615 (52.3%)	7,260 (53.0%)	7,817 (51.6%)	4,855 (52.6%)	1,518 (58.3%)	973 (58.5%)	946 (60.4%)	621 (61.1%)
Male	10,557 (47.5%)	6,425 (46.9%)	7,314 (48.3%)	4,369 (47.3%)	1,084 (41.6%)	688 (41.4%)	619 (39.5%)	394 (38.8%)
Other	30 (0.1%)	15 (0.1%)	15 (0.1%)	8 (0.1%)	1 (0.0%)	1 (0.1%)	1 (0.1%)	1 (0.1%)
Missing data	4,052	2,564	4,465	2,705	369	247	318	229
Age (years), median (IQR)	38 (23, 57)	42 (25, 60)	35 (20, 51)	38 (21, 54)	48 (36, 63)	52 (38, 65)	45 (34, 59)	47 (36, 60)
Age group (years), n (%)								
12-15	3,581 (13.6%)	2,156 (13.3%)	3,313 (16.9%)	1,972 (16.5%)	3 (0.1%)	0 (0.0%)	2 (0.1%)	0 (0.0%)
16-19	1,789 (6.8%)	948 (5.8%)	1,540 (7.9%)	816 (6.8%)	79 (2.7%)	42 (2.2%)	46 (2.5%)	26 (2.1%)
20-29	3,902 (14.9%)	1,994 (12.3%)	3,000 (15.3%)	1,507 (12.6%)	341 (11.5%)	165 (8.7%)	234 (12.5%)	125 (10.1%)
30-39	4,314 (16.4%)	2,469 (15.2%)	3,499 (17.9%)	1,996 (16.7%)	580 (19.6%)	322 (16.9%)	407 (21.7%)	255 (20.6%)
40-49	3,567 (13.6%)	2,233 (13.7%)	2,870 (14.6%)	1,782 (14.9%)	530 (17.9%)	330 (17.4%)	394 (21.0%)	267 (21.6%)
50-59	3,287 (12.5%)	2,228 (13.7%)	2,523 (12.9%)	1,742 (14.6%)	529 (17.9%)	348 (18.3%)	351 (18.7%)	248 (20.0%)
60-69	2,966 (11.3%)	2,144 (13.2%)	1,862 (9.5%)	1,377 (11.5%)	501 (16.9%)	374 (19.7%)	263 (14.0%)	187 (15.1%)
70-79	2,143 (8.2%)	1,592 (9.8%)	771 (3.9%)	574 (4.8%)	320 (10.8%)	260 (13.7%)	145 (7.7%)	107 (8.6%)
80+	692 (2.6%)	491 (3.0%)	224 (1.1%)	164 (1.4%)	79 (2.7%)	60 (3.2%)	34 (1.8%)	23 (1.9%)
Missing data	13	9	9	7	10	8	8	7
Indigenous status, n (%)								
Aboriginal and/or Torres Strait Islander	733 (2.9%)	419 (2.6%)	529 (2.8%)	332 (2.8%)	61 (2.1%)	31 (1.6%)	41 (2.2%)	27 (2.2%)
Non-indigenous	24,907 (97.1%)	15,719 (97.4%)	18,652 (97.2%)	11,395 (97.2%)	2,858 (97.9%)	1,872 (98.4%)	1,804 (97.8%)	1,213 (97.8%)
Missing data	614	126	430	210	53	6	39	5

* Proportions exclude missing data. Only day 8 surveys where the respondent had also answered a day 3 survey were included.
IQR = interquartile range

Table S4. Any adverse event following COVID-19 vaccination, as reported in the AusVaxSafety day 3 COVID-19 vaccine safety survey, September 2021 to September 2023, by vaccine, dose number, and respondent characteristics*

	Spikevax				Nuvaxovid			
	Dose 1		Dose 2		Dose 1		Dose 2	
Characteristic	Any adverse event n/N (%)	aOR (95% Crl)	Any adverse event n/N (%)	aOR (95% Crl)	Any adverse event n/N (%)	aOR (95% Crl)	Any adverse event n/N (%)	aOR (95% Crl)
All respondents	10,930/26,224 (41.7%)		12,573/19,596 (64.2%)		1,087/2,971 (36.6%)		1,078/1,883 (57.2%)	
Sex/Age[#]								
Male 12-15 years	426/1,329 (32.1%)	0.89 (0.76, 1.03)	754/1,177 (64.1%)	1.28 (1.09, 1.50)	0/1 (0.0%)	1.28 (0.20, 4.38)		1.26 (0.23, 4.00)
Male 16-19 years	184/649 (28.4%)	0.72 (0.59, 0.87)	299/551 (54.3%)	0.89 (0.73, 1.08)	6/27 (22.2%)	0.72 (0.26, 1.50)	9/18 (50.0%)	0.88 (0.33, 1.94)
Male 20-29 years	555/1,581 (35.1%)	1.00	616/1,075 (57.3%)	1.00	42/116 (36.2%)	1.00	44/83 (53.0%)	1.00
Male 30-39 years	712/1,678 (42.4%)	1.25 (1.09, 1.44)	830/1,267 (65.5%)	1.42 (1.21, 1.66)	80/187 (42.8%)	1.36 (0.89, 2.02)	66/105 (62.9%)	1.46 (0.88, 2.33)
Male 40-49 years	576/1,444 (39.9%)	1.12 (0.97, 1.29)	670/1,105 (60.6%)	1.13 (0.96, 1.33)	73/188 (38.8%)	1.12 (0.72, 1.66)	72/124 (58.1%)	1.26 (0.78, 1.97)
Male 50-59 years	524/1,385 (37.8%)	0.94 (0.81, 1.09)	584/1,019 (57.3%)	0.90 (0.76, 1.06)	65/199 (32.7%)	0.88 (0.56, 1.31)	60/131 (45.8%)	0.91 (0.56, 1.41)
Male 60-69 years	446/1,292 (34.5%)	0.68 (0.58, 0.80)	370/733 (50.5%)	0.75 (0.62, 0.89)	44/203 (21.7%)	0.58 (0.36, 0.88)	39/91 (42.9%)	0.69 (0.40, 1.11)
Male 70-79 years	248/904 (27.4%)	0.41 (0.34, 0.50)	119/309 (38.5%)	0.54 (0.41, 0.68)	22/128 (17.2%)	0.40 (0.22, 0.66)	21/53 (39.6%)	0.67 (0.34, 1.18)
Male ≥80 years	61/289 (21.1%)	0.29 (0.21, 0.38)	21/77 (27.3%)	0.33 (0.20, 0.52)	1/30 (3.3%)	0.18 (0.05, 0.45)	0/10 (0.0%)	0.20 (0.04, 0.56)
Female 12-15 years	493/1,427 (34.5%)	1.03 (0.89, 1.19)	759/1,209 (62.8%)	1.22 (1.04, 1.42)	0/2 (0.0%)	0.95 (0.16, 2.92)	-	1.27 (0.21, 4.27)
Female 16-19 years	276/765 (36.1%)	1.01 (0.85, 1.21)	306/560 (54.6%)	0.90 (0.74, 1.08)	6/33 (18.2%)	0.93 (0.39, 1.81)	5/16 (31.3%)	0.49 (0.16, 1.13)

Characteristic	Spikevax				Nuvaxovid			
	Dose 1		Dose 2		Dose 1		Dose 2	
	Any adverse event n/N (%)	aOR (95% Crl)	Any adverse event n/N (%)	aOR (95% Crl)	Any adverse event n/N (%)	aOR (95% Crl)	Any adverse event n/N (%)	aOR (95% Crl)
Female 20-29 years	740/1,499 (49.4%)	1.76 (1.52, 2.04)	849/1,156 (73.4%)	2.01 (1.68, 2.39)	93/170 (54.7%)	2.45 (1.55, 3.65)	65/108 (60.2%)	1.69 (1.01, 2.70)
Female 30-39 years	1,056/1,884 (56.1%)	2.15 (1.89, 2.46)	1,100/1,439 (76.4%)	2.41 (2.05, 2.83)	150/303 (49.5%)	1.81 (1.21, 2.58)	164/237 (69.2%)	1.96 (1.28, 2.92)
Female 40-49 years	912/1,569 (58.1%)	2.24 (1.94, 2.57)	907/1,157 (78.4%)	2.53 (2.11, 3.01)	138/261 (52.9%)	1.98 (1.32, 2.85)	145/203 (71.4%)	2.22 (1.42, 3.34)
Female 50-59 years	826/1,496 (55.2%)	1.82 (1.57, 2.09)	719/1,006 (71.5%)	1.68 (1.40, 2.00)	103/261 (39.5%)	1.15 (0.77, 1.65)	99/154 (64.3%)	1.73 (1.08, 2.67)
Female 60-69 years	716/1,459 (49.1%)	1.25 (1.07, 1.44)	531/826 (64.3%)	1.29 (1.07, 1.55)	87/263 (33.1%)	0.94 (0.62, 1.37)	70/137 (51.1%)	0.97 (0.61, 1.49)
Female 70-79 years	427/1,140 (37.5%)	0.66 (0.55, 0.77)	186/359 (51.8%)	0.90 (0.71, 1.14)	37/178 (20.8%)	0.53 (0.32, 0.82)	26/73 (35.6%)	0.57 (0.31, 0.97)
Female ≥80 years	107/373 (28.7%)	0.44 (0.34, 0.56)	37/104 (35.6%)	0.43 (0.28, 0.63)	8/44 (18.2%)	0.44 (0.18, 0.83)	4/16 (25.0%)	0.33 (0.11, 0.76)
Indigenous status								
Aboriginal & Torres Strait Islander	275/732 (37.6%)	0.79 (0.67, 0.92)	287/529 (54.3%)	0.59 (0.49, 0.70)	21/61 (34.4%)	0.83 (0.46, 1.38)	20/41 (48.8%)	0.77 (0.40,1.38)
Non-Aboriginal	10,392/24,883 (41.8%)	1.00	12,016/18,637 (64.5%)	1.00	1,037/2,858 (36.3%)	1.00	1,031/1,804 (57.2%)	1.00
Clinic type								
General Practice - Opt out	2,658/5,598 (47.5%)	1.00	242/538 (45.0%)	1.00	332/1,074 (30.9%)	1.00	278/470 (59.1%)	1.00
State Health - Opt out	2,862/7,006 (40.9%)	0.58 (0.52, 0.64)	3,446/5,487 (62.8%)	1.69 (1.37, 2.06)	477/1,102 (43.3%)	1.29 (1.05, 1.56)	389/691 (56.3%)	0.78 (0.59, 1.00)
State Health - Opt in	14/33 (42.4%)	0.82 (0.38, 1.56)	2/6 (33.3%)	0.67 (0.15, 1.96)	5/5 (100.0%)	4.83 (0.87,16.43)	4/7 (57.1%)	1.08 (0.25, 3.24)

Characteristic	Spikevax				Nuvaxovid			
	Dose 1		Dose 2		Dose 1		Dose 2	
	Any adverse event n/N (%)	aOR (95% CrI)	Any adverse event n/N (%)	aOR (95% CrI)	Any adverse event n/N (%)	aOR (95% CrI)	Any adverse event n/N (%)	aOR (95% CrI)
Pharmacy - Opt out	5,369/13,536 (39.7%)	0.57 (0.52, 0.62)	8,867/13,544 (65.5%)	1.87 (1.54, 2.25)	270/774 (34.9%)	1.00 (0.80, 1.24)	398/698 (57.0%)	0.87 (0.67, 1.10)
Pharmacy - Opt in	17/30 (56.7%)	1.33 (0.48, 3.04)	10/15 (66.7%)	1.25 (0.29, 3.69)	-	1.68 (0.14, 7.38)	-	1.64 (0.15, 6.81)
Aboriginal Health Service - Opt out	8/18 (44.4%)	0.92 (0.35, 1.95)	3/3 (100.0%)	3.43 (0.52,12.33)	3/16 (18.8%)	0.80 (0.23, 1.90)	9/16 (56.3%)	1.03 (0.35, 2.44)
State/Territory								
ACT	279/594 (47.0%)	1.00 (0.83, 1.19)	126/178 (70.8%)	1.30 (0.92, 1.81)	0/1 (0.0%)	1.13 (0.12, 4.27)	-	1.70 (0.14, 7.29)
NSW	2,057/4,489 (45.8%)	1.00	1,545/2,455 (62.9%)	1.00	224/675 (33.2%)	1.00	199/377 (52.8%)	1.00
NT	12/22 (54.5%)	0.97 (0.30, 2.43)	9/12 (75.0%)	2.71 (0.50, 8.64)	-	1.66 (0.14, 7.18)	-	1.66 (0.14, 7.11)
QLD	1,358/3,358 (40.4%)	0.87 (0.79, 0.96)	1,393/2,113 (65.9%)	1.01 (0.88, 1.14)	84/280 (30.0%)	0.88 (0.63, 1.20)	101/171 (59.1%)	1.32 (0.87, 1.92)
SA	493/1,243 (39.7%)	0.87 (0.76, 1.00)	591/897 (65.9%)	0.99 (0.83, 1.16)	35/101 (34.7%)	1.11 (0.68, 1.70)	49/88 (55.7%)	1.16 (0.70, 1.83)
TAS	164/420 (39.0%)	0.88 (0.71, 1.08)	167/296 (56.4%)	0.66 (0.51, 0.85)	9/29 (31.0%)	0.92 (0.38, 1.83)	11/19 (57.9%)	1.38 (0.54, 2.93)
VIC	4,745/11,619 (40.8%)	0.93 (0.84, 1.01)	6,549/10,332 (63.4%)	0.90 (0.81, 1.00)	221/597 (37.0%)	0.98 (0.76, 1.25)	356/611 (58.3%)	1.26 (0.95, 1.64)
WA	1,820/4,476 (40.7%)	0.91 (0.83, 1.00)	2,190/3,310 (66.2%)	0.99 (0.89, 1.11)	514/1,288 (39.9%)	1.28 (1.04, 1.56)	362/617 (58.7%)	1.33 (1.01, 1.74)
Anaphylaxis history								
No	10,602/25,606 (41.4%)	1.00	12,231/19,131 (63.9%)	1.00	1,007/2,832 (35.6%)	1.00	1,006/1,780 (56.5%)	1.00

Characteristic	Spikevax				Nuvaxovid			
	Dose 1		Dose 2		Dose 1		Dose 2	
	Any adverse event n/N (%)	aOR (95% Crl)	Any adverse event n/N (%)	aOR (95% Crl)	Any adverse event n/N (%)	aOR (95% Crl)	Any adverse event n/N (%)	aOR (95% Crl)
Yes	328/618 (53.1%)	1.76 (1.27, 2.37)	342/465 (73.5%)	1.50 (0.97, 2.26)	80/139 (57.6%)	2.86 (1.56, 4.85)	72/103 (69.9%)	1.72 (0.81, 3.26)
Underlying medical condition								
None	9,032/22,609 (39.9%)	1.00	11,118/17,497 (63.5%)	1.00	833/2,373 (35.1%)	1.00	895/1,565 (57.2%)	1.00
Any	1,898/3,615 (52.5%)	-	1,455/2,099 (69.3%)	-	254/598 (42.5%)	-	183/318 (57.5%)	-
Coronary heart disease	225/510 (44.1%)	1.27 (1.03, 1.55)	113/208 (54.3%)	0.83 (0.60, 1.12)	24/86 (27.9%)	1.05 (0.60, 1.70)	14/35 (40.0%)	0.78 (0.35, 1.52)
Poorly controlled hypertension	147/304 (48.4%)	1.21 (0.93, 1.55)	82/120 (68.3%)	1.52 (0.99, 2.27)	15/50 (30.0%)	0.75 (0.36, 1.36)	10/19 (52.6%)	1.53 (0.55, 3.44)
Diabetes	370/828 (44.7%)	1.03 (0.88, 1.21)	266/437 (60.9%)	0.93 (0.75, 1.15)	34/99 (34.3%)	1.31 (0.79, 2.02)	18/51 (35.3%)	0.54 (0.26, 0.95)
Chronic lung disease	163/317 (51.4%)	1.31 (1.02, 1.66)	95/152 (62.5%)	1.03 (0.69, 1.45)	21/47 (44.7%)	1.58 (0.79, 2.77)	20/35 (57.1%)	1.31 (0.58, 2.56)
Obesity	199/346 (57.5%)	1.34 (1.04, 1.69)	132/175 (75.4%)	1.55 (1.04, 2.26)	19/53 (35.8%)	0.91 (0.46, 1.62)	14/30 (46.7%)	0.93 (0.41, 1.86)
Chronic kidney failure	35/82 (42.7%)	0.89 (0.53, 1.40)	26/48 (54.2%)	1.01 (0.50, 1.84)	5/12 (41.7%)	1.42 (0.36, 3.72)	4/7 (57.1%)	1.55 (0.35, 4.47)
Chronic liver disease	41/68 (60.3%)	1.85 (1.06, 3.04)	29/40 (72.5%)	1.65 (0.78, 3.18)	2/7 (28.6%)	0.79 (0.17, 2.21)	6/7 (85.7%)	3.58 (0.75,11.42)
Non-haematological malignancies diagnosed in past 12 months	71/168 (42.3%)	1.11 (0.77, 1.54)	45/75 (60.0%)	1.00 (0.59, 1.60)	2/18 (11.1%)	0.45 (0.11, 1.14)	9/16 (56.3%)	1.69 (0.56, 3.97)
Haematological malignancies	50/106 (47.2%)	1.36 (0.87, 1.98)	47/67 (70.1%)	1.70 (0.94, 2.92)	2/9 (22.2%)	0.77 (0.17, 2.17)	2/4 (50.0%)	1.34 (0.23, 4.36)

Characteristic	Spikevax				Nuvaxovid			
	Dose 1		Dose 2		Dose 1		Dose 2	
	Any adverse event n/N (%)	aOR (95% CrI)	Any adverse event n/N (%)	aOR (95% CrI)	Any adverse event n/N (%)	aOR (95% CrI)	Any adverse event n/N (%)	aOR (95% CrI)
diagnosed in the past five years								
Currently receiving chemotherapy or radiotherapy	23/60 (38.3%)	0.97 (0.51, 1.66)	26/35 (74.3%)	2.09 (0.86, 4.37)	1/6 (16.7%)	0.91 (0.15, 2.95)	1/5 (20.0%)	0.64 (0.11, 2.02)
Organ transplant recipient on immune suppressive therapy	34/63 (54.0%)	1.42 (0.82, 2.33)	27/51 (52.9%)	0.75 (0.39, 1.30)	4/7 (57.1%)	2.37 (0.56, 6.81)	1/2 (50.0%)	1.23 (0.15, 4.64)
Bone marrow transplant recipient in past two years	1/4 (25.0%)	0.70 (0.12, 2.26)	4/7 (57.1%)	0.89 (0.20, 2.67)	2/3 (66.7%)	1.86 (0.29, 6.45)	1/1 (100.0%)	2.22 (0.24, 9.21)
Neurological condition	106/200 (53.0%)	1.23 (0.90, 1.64)	65/94 (69.1%)	1.17 (0.72, 1.81)	15/35 (42.9%)	1.19 (0.56, 2.22)	13/25 (52.0%)	0.85 (0.33, 1.85)
Chronic inflammatory conditions	366/629 (58.2%)	1.57 (1.32, 1.86)	311/405 (76.8%)	1.61 (1.24, 2.06)	53/119 (44.5%)	1.34 (0.87, 2.00)	38/72 (52.8%)	0.84 (0.49, 1.37)
Primary or acquired immunodeficiency	40/66 (60.6%)	1.74 (1.00, 2.87)	38/51 (74.5%)	1.44 (0.72, 2.65)	6/12 (50.0%)	2.24 (0.68, 5.57)	2/5 (40.0%)	0.86 (0.16, 2.63)

aOR = adjusted odds ratio; CrI = credible interval

* aORs not reported for categories where no data is reported

Proportions are calculated from individuals identifying as male or female, not accounting for where sex is missing (18.1%) and for the very small sample of those identifying as sex = Other (0.1%)/sex = Other and reported any AEFI (0.05%)
